# Association between Family Functioning, Child Emotional and Behavioral Problems, and Parental Stress during the COVID-19 Pandemic in Thailand

**DOI:** 10.3390/bs14040270

**Published:** 2024-03-24

**Authors:** Sawitree Jetiyanuwat, Suttipong Kawilapat, Assawin Narkpongphun, Pichaya Pojanapotha

**Affiliations:** 1 Bangkok Rayong Hospital, Rayong 21000, Thailand; mashipraew@gmail.com; 2 Department of Psychiatry, Faculty of Medicine, Chiang Mai University, Chiang Mai 50200, Thailand; suttipong.kawilapat@cmu.ac.th (S.K.); assawin.nark@cmu.ac.th (A.N.)

**Keywords:** COVID-19, children, emotional problem, behavioral problem, screen time, SDQ, ST-5, SCORE-15

## Abstract

The COVID-19 pandemic has had a huge impact on people of all ages, especially children. This is a cross-sectional study in Thailand to explore the emotional and behavioral problems of school-aged children and associated factors during the lockdown. An online survey was conducted with 942 parents of school-age children. Strengths and Difficulties Questionnaire (SDQ) scores showed that total difficulties and all subscale difficulties (hyperactivity, conduct problems, peer problems, and emotional problems) were increased, whereas prosocial behaviors were decreased in the pandemic period. The factors significantly associated with higher parental stress were higher emotional and peer problems after the COVID-19 outbreak, high family difficulty, and sleep problems. Sleep problems were associated with all children’s difficulties, except prosocial behavior. High score in family difficulty subscale was associated with increased emotional problems, whereas poor family communication was associated with increased hyperactivity. Appetite change was negatively associated with parental stress and some children’s difficulties. Higher household income, family time, physical activities, and recreational activities were associated with a decreased level of some difficulties and family functioning problems, but positively with an increase in the prosocial behavior of children. Additionally, higher screen time was associated with a higher level of hyperactivity, conduct problems, and poor family communication. This study demonstrated that Thai children were at high risk of developing mental health problems during the pandemic lockdown. We suggest that intervention to promote physical activities and reduce screen time is needed. Moreover, efficient monetary policy is urgently required. The limitations here include a recall bias with no baseline to compare and a potential selection bias due to parental selection and a webpage announcement.

## 1. Introduction

The coronavirus disease 2019 (COVID-19) first appeared in China in late 2019 and then rapidly spread around the world, becoming the most severe global health crisis in 2020–2022. The World Health Organization (WHO) declared the COVID-19 outbreak a global pandemic on 11 March 2020 [1,2]. Recently, more than 700 million people were infected, and total deaths worldwide were more than 7 million [3].

During the long-term COVID-19 pandemic, quarantine and lockdown policies were implemented in many countries, including Thailand [4]. Consequently, family members spent more time together in their houses, and they were forced to regulate themselves to the new normal, with uncertainty and fewer coping strategies. Daily routines needed reorganization, with fewer physical activities and increased screen time in all age groups, especially school-aged children [5]. As a result, sleep disturbances, appetite changes, and impairments in social interactions were demonstrated [6]. A number of studies showed an increased prevalence of mental health problems in children and adolescents, such as depression, anxiety, irritability, inattention, and child maltreatment [7,8,9,10,11,12]. A systematic review showed that school closure and social lockdown during the COVID-19 pandemic were associated with poor health behavior (such as lower physical activities and higher screen time) and mental health problems among children and adolescents [13]. Okuyama’s review showed a correlation between physical activity and mental health [14].

In addition to the child and adolescent mental health problems, many studies demonstrated the effect of the COVID-19 pandemic on parental stress and family well-being. Chen et al. reported parental burnout and mental health problems among Chinese parents, especially in families with younger children. It was shown that family structure and family function moderated the relationship between burnout and mental health [15]. An online survey in the United Arab Emirates showed that higher parental stress and lower child well-being were associated with pandemic-related stressors, and family relationship was the mediator [16]. Dayton’s work showed that parental caregiving strain was a risk factor associated with child anxiety, and income loss was associated with sleep disturbances [17].

In Thailand, it was reported that more than half of families with primary school children were likely to be at a moderate or poor level of family health during the COVID-19 pandemic [18]. Interestingly, the online survey in Thai families showed that the general family functioning, strength, and communication subscales of Thai families after the onset of COVID-19 pandemic were significantly improved compared to the pre-pandemic period, but perceived family happiness was decreased during the pandemic. The study showed that the loss of close persons from COVID-19 and having financial problems were associated with family happiness [19].

Although some studies mentioned family functioning and parental stress during the COVID-19 pandemic, few studies related these concerns to child emotional and behavioral problems. This study aimed to examine the association between parental stress, family functioning, child emotional and behavioral problems, and associated factors during the COVID-19 pandemic lockdown in Thailand. We hypothesized that some independent variables, such as financial problems, sleep problems, screen time, and physical activities, affect each dimension of family functioning (strength and adaptability, overwhelmed by difficulties, and disrupted communication), SDQ score change (before and during the COVID-19 pandemic), and parental stress. In addition, we proposed that family functioning is correlated with parental stress and SDQ score changes.

## 2. Materials and Methods

### 2.1. Participants

Participants were the main caregivers of elementary students (Grade 1–6) in Thailand and were able to read and write the Thai language fluently. We set the prevalence of children with emotional and behavioral problems for sample size calculation at 50%, which yields the highest sample size. Under the assumptions of a 95% confidence interval (CI) and a 5% precision error, the sample size was calculated using the following equation:

(1) $$n=\frac{\left({Z_{\alpha /2})}^{2}P(1-P\right)}{d^{2}}=\frac{{\left(1.96\right)}^{2}(0.50)(1-0.50)}{{\left(0.05\right)}^{2}}=384.16\;\approx 385$$

Three hundred and eighty-five parents of school-aged children was the minimum required sample size for the current study. To account for sampling error, we increased the sample size by 10%. Therefore, the adjusted required sample size was 424 participants.

### 2.2. Procedures

A cross-sectional online survey was conducted in June 2020, via a public online platform (Facebook pages providing information about parenting). All participants voluntarily answered an anonymous online questionnaire and indicated their informed consent. The survey took approximately 10–20 min to complete. If participants had more than one child, they were asked to report on only one child.

### 2.3. Measurements

Demographic data: the first part of the questionnaire collected data on respondents (relationship with children, educational level, income, occupation), the child’s information (sex, age, and class level), the direct consequences of COVID-19, and the child’s daily activities (such as eating, sleeping, physical activities, etc.).

The Strengths and Difficulties Questionnaire (SDQ): The SDQ Thai improved version was used in this study. It consists of 25 questions about positive and negative behaviors of children and adolescents that can be allocated to 5 subscales of 5 items: emotional, conduct, hyperactivity, peer problems, and prosocial behavior [20]. In brief, each item was scored on a 3-point scale with 0 = not true, 1 = somewhat true, and 2 = certainly true. Higher scores on the prosocial behavior subscale reflected strengths, whereas higher scores on emotional, conduct, hyperactivity, and peer problems reflected difficulties. A total score of these four subscales revealed total difficulties, ranging from 0–40. A higher score reflected a higher risk of developing a mental health problem. The normal range of total difficulties score is 0–15. A score of more than 19 was considered abnormal, whereas 16–18 points were considered as borderline. Prosocial behavior scores were categorized as normal and abnormal [20,21,22].

SCORE-15: The 15-item Systemic Clinical Outcome and Routine Evaluation is a self-report questionnaire. It measures family functioning in three domains: family strengths, difficulty, and communication. It includes 15 Likert-scale (range 1–5) items. The greater SCORE-15 index reflects poorer family functioning [23]. The SCORE-15 Thai version was developed and tested for its psychometric properties. The internal reliability and convergent validity in the Thai population were acceptable [24].

ST-5: Parental stress was assessed by the stress questionnaire developed by the Department of Mental Health, Ministry of Public Health of Thailand. It is a self-report questionnaire composed of five Likert-scale questions ranging from 0 (never or rarely) to 3 (always). The total score ranges from 0 to 15, as follows: 0–4 = mind stress, 5–7 = moderate stress, 8–9 = high stress, and 10–15 = very high stress [25].

### 2.4. Statistical Analysis

The demographics and characteristics of caregivers who responded to the survey and their children were described using frequencies and percentages for categorical variables and as means and standard deviations (SDs) for continuous variables. Fisher’s exact test and independent t-test were used to compare the differences in strengths and difficulties among children before and after the spread of COVID-19 for categorical variables and continuous variables, respectively. The association between demographics, family functioning, changes in the SDQ score of children after the spread of COVID-19, and parental stress were preliminary examined using the Pearson correlation coefficient. All the paths between variables with significant associations in the correlation analysis were included in the path analysis. According to the path analysis, the goodness of fit statistics were determined by the relative chi-square (χ^2^/df), which was no more than 2 [26] and had a *p*-value of >0.05 [27]. Additionally, the comparative fit index (CFI) and the Tucker–Lewis index (TLI) were >0.95, while the root mean square error index of approximation (RMSEA) and standardized root mean square residual (SRMR) were <0.05 [28]. Variables with a *p*-value < 0.05 were considered statistically significant. All analyses were performed using Stata version 17.

## 3. Results

### 3.1. Demographic Data of Participants

Of the 942 participants who responded to this survey, the majority of respondents were the mothers of the children in question (88.9%). Most respondents graduated with a bachelor’s degree or higher. Household incomes varied from less than USD 300 to more than USD 3000 per month. The number of boys in care was slightly higher than girls (52.1% versus 47.9%), with an average age of 8.54 years. Most of their children attended private schools (60.2%) rather than governmental schools (27.9%). Some children (16%) had psychiatric and/or neurodevelopmental disorders, mostly ADHD (Table 1).

### 3.2. Effect of the COVID-19 Pandemic on Children’s Daily Activities

Table 2 shows that only a few families in this study were infected (1 child and 1 parent) or quarantined due to being part of a high-risk group (13 children and 21 parents). A total of 25.8% of the children reported new or worse sleep problems during the COVID-19 outbreak. Most of them had physical activities for less than one hour per day and took less than two hours a day for recreational activities. A total of 36.1% of parents spent less than an hour a day with their children. The majority of parents reported that their children spent more time on screens during the COVID-19 outbreak, and only 27.6% of them were available for all-time supervision during the screen time (Table 2).

### 3.3. Emotional and Behavioral Problems Developed Due to the COVID-19 Pandemic

**Figure 1:**
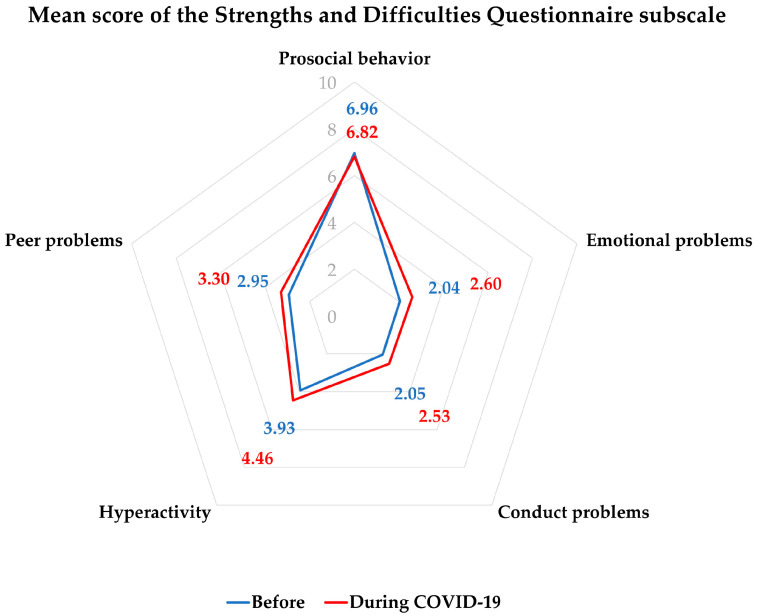
Mean score of the Strengths and Difficulties Questionnaire subscale, before and during the COVID-19 pandemic.

**Figure 2:**
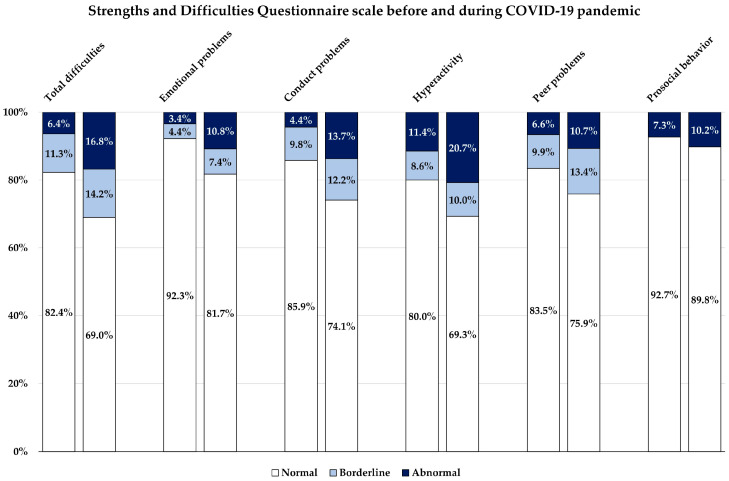
Comparison of the Strengths and Difficulties Questionnaire subscale, before and during the COVID-19 pandemic.

The mean SDQ score comparing before and during the COVID-19 pandemic is shown in Table 3. According to the radar diagram, except for prosocial behavior, hyperactivity was highest, followed by peer problems, emotional problems, and conduct problems, respectively. The patterns of the mean SDQ scores of all subscales were quite similar between before and during the COVID-19 pandemic (Figure 1). However, total difficulties and all subscale scores (emotional, conduct, hyperactivity, and peer problems) were significantly increased, while prosocial behavior was significantly decreased. The total difficulties and each subscale score were categorized into normal, borderline, and abnormal groups, while the prosocial behavior score was categorized into normal and abnormal groups (Table 3). It is clearly shown in Figure 2 that the abnormal groups in all subscales increased after the outbreak of COVID-19.

### 3.4. Association between Parental Stress, Family Functioning, and Children’s Strengths and Difficulties during the COVID-19 Pandemic

According to correlation analysis, all family functioning subscales and difficulties subscales of children (except prosocial behavior) were associated with parental stress. Family functioning subscales were also associated with several difficulties for children. Several demographic variables were significantly associated with parental stress, family functioning, and children’s strengths and difficulties, including household income, child’s age, previous psychiatric diagnosis, sleep problems, appetite changes, physical activities, recreational activities, family time, and screen time (Table S1, Supplementary Materials). All the paths with significant correlations of all variables were included as the hypothetical model for path analysis.

The results of the path analysis in Table 4 showed that all of the fit indices of the hypothetical model were adequate, namely SRMR = 0.019, RMSEA = 0.027, CFI = 0.995, and TLI = 0.985. The higher emotional problems (*β* = 0.073), higher peer problems (*β* = 0.0795), increased family difficulty score (*β* = 0.2597), and sleep problems (*β* = 0.2267) were associated with a higher level of parental stress, whereas a child’s appetite change was associated with a lower level of parental stress (*β* = −0.655). An increased family difficulty score (*β* = 0.0812) and sleep problems (*β* = 0.1969) were also associated with increased emotional problems during the COVID-19 pandemic, whereas physical activities at least one hour per day were associated with a decrease in score difference (*β* = −0.0732). Sleep problems (*β* = 0.1284) and screen time of three hours per day or more (*β* = 0.0927) were associated with increased conducted problems, whereas appetite changes (*β* = −0.0894) and family time of at least one hour per day (*β* = −0.0812) were associated with decreased score differences. Family strength (*β* = 0.0707), sleep problems (*β* = 0.1515), and screen time of three hours per day or more (*β* = 0.0821) were associated with higher hyperactivity differences, whereas family time of at least one hour per day (*β* = −0.1037) could reduce the hyperactivity differences. Sleep problems of children were also associated with increased peer problems (*β* = 0.0944), whereas appetite changes (*β* = −0.0645) and physical activities at least one hour per day (*β* = −0.1118) were associated with a lower difference. The factors associated with a higher prosocial behavior level were high income (>900 USD per month) (*β* = 0.0960), as well as physical activities (*β* = 0.0918) and recreational activities (*β* = 0.0896) for at least one hour per day. Interestingly, a child’s age was shown to have a negative association with prosocial behavior change (*β* = −0.0916).

**Figure 3:**
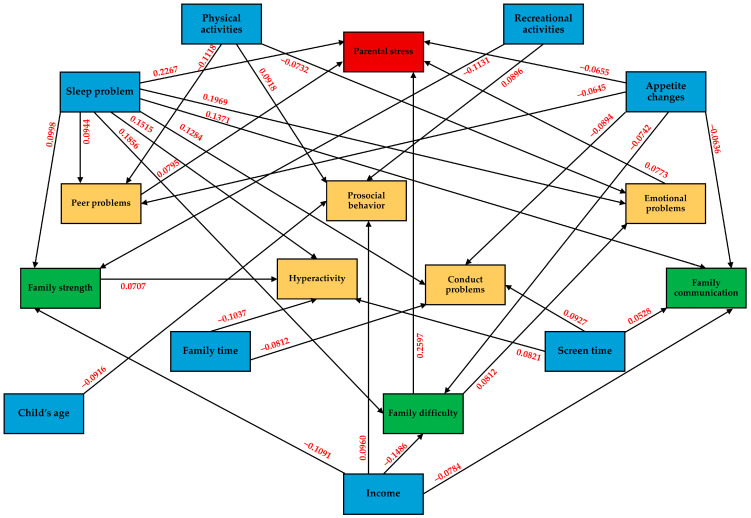
Path diagram for factors with significant associations. Blue = demographics; green = family functioning; yellow = child emotional and behavioral difficulties; red = parental stress.

According to family functioning, the demographic associated with poorer family strength (higher family strength score) was sleep problems (*β* = 0.0998), whereas high income (*β* = −0.1091) and recreational activities for at least one hour per day (*β* = −0.1131) were associated with better family strength. Sleep problems (*β* = 0.1856) were also associated with a higher family difficulty score, whereas high income (*β* = −0.1486) and appetite changes (*β* = −0.0742) were associated with a lower score. The factors associated with poorer family communication were sleep problems (*β* = 0.1371) and screen time of three hours per day or more (*β* = 0.0528), whereas high income (*β* = −0.0784) and appetite changes (*β* = −0.0636) were associated with better family communication (Table 4). The path diagram of significant associations is illustrated in Figure 3.

## 4. Discussion

This cross-sectional study confirmed that the daily routine of children was disrupted during the COVID-19 pandemic; 25.8% of the children developed sleep problems, and 89.1% showed appetite change. Most of them had less than an hour of physical activity per day. Screen time was obviously increased for 86.1% of them. Our results were consistent with previous studies [5,29,30,31,32,33]. The comparison of the SDQ subscales showed increased emotional and behavioral problems during the COVID-19 pandemic. It also clearly demonstrated that the proportion of children whose SDQ scores were within the normal range significantly decreased in all subscales. This is in agreement with Viner’s systematic review about mental health of children and adolescents during the first COVID-19 wave [13]. Several studies during the long-term pandemic also demonstrated the global burden of mental health problems among children and adolescents [34].

Despite numerous studies that have shown child and parental mental health problems, only a few studies have been carried out in Thailand. Our data were collected in June 2020, when there were a cumulative 3000–4000 confirmed COVID-19 cases in Thailand. The emergency decree was declared on 26 March, and a curfew went into effect on 3 April 2020. Social measures and a full-scale national lockdown were implemented. New cases in the country occurred in the plateau phase in late May 2020; however, the worldwide situation was still in crisis, and Thailand was still under a strict lockdown during that time. At that moment, Thai children and parents needed to cope with uncertain school situations. Most nursery and primary schools postponed the start of the semester, which normally used to be in May. Some schools compensated by online studying; some schools tried to let students go to school on alternate days or weeks. Teachers tended to assign more homework to compensate for less onsite studying. Consequently, parents and children were stressed by uncertain and unfamiliar situations. Parents were forced to arrange childcare during the quarantine measure whether they were ready or not. Meanwhile, they also faced challenges in their own work. Some parents had to work from home, while some were at risk of being unemployed or having a decreased income. The economic security of families was threatened [35].

Figure 3 shows that increased screen time was correlated with more hyperactivity problems, conduct problems, and poor family communication. It has been shown in many studies that screen time was associated with child well-being and child development [36,37,38]. It is not surprising that screen time was found to be associated with poor family communication since it was associated with children’s social isolation and hindered opportunities for social interaction [39]. Hence, this leads to a decreased ability to cope with emotional stress in daily life [40]. However, managing screen time during the pandemic is challenging. When outdoor activities were restricted, media use was the most common coping strategy for children and parents. In this study, 14.9% spent more than seven hours per day in front of electronic device screen and 22.6% had a screen time of five to seven hours per day. In addition, 84.2% of the children had online classes, and 33.3% of them had to study online for more than three hours per day. Meanwhile, one-third of their parents reported no or only occasional supervision during their children’s screen time. Werling’s work showed that there would be some children who could not return to their pre-Covid screen time even after the lockdown [41].

As shown in Figure 3, physical activities were correlated with changing peer problems, changing emotional problems, and changing prosocial behavior. Our results supported previous evidence that showed a close relationship between physical activities and psychological health during the COVID-19. Less physical activities caused mental health problems; meanwhile, depression and anxiety are also causes of fatigue, exhaustion, and low energy, which lead to less physical activities [42]. Physical activities in school-age children usually mean social interactions, either with peers or caregivers. Therefore, it is understandable that less physical activity was correlated with peer problems. Moreover, it was demonstrated in junior high school students that physical activities improved prosocial behavior [43,44]. The systematic review also showed that sports activities influence the prosocial behavior of children and adolescents [44]. Interestingly, we found that there was a higher difference in the prosocial behavior score in younger children than in older children during the COVID-19 pandemic. This could imply that the pandemic was more impactful for younger children. Taken together, the screen time problem and physical activities are worth worrying about and should be mentioned in child mental health policies. Safe outdoor activities, especially for younger children, should be easier to access in both geographical and economic terms. In addition, there should be more creative activity choices for families.

Concerning the economic insecurity mentioned above, we found that high-income families (more than 900 USD per month) are associated with a higher level of prosocial behavior compared to those with lower incomes. Moreover, path analysis showed that lower income correlated with poorer family functioning on all subscales. Those problems were then associated with some routine changes or SDQ subscales changes, which finally led to parental stress. This result is similar to a longitudinal study about the psychosocial status and mental health of children in Europe [45]. An online survey in parents in the U.S. showed that the majority of them reported at least one symptom of parental burnout at the beginning of COVID-19. Among those families, children from low-income families showed more stress-related behaviors, such as mood swings, nightmares, etc., compared to those from high-income families [46]. In Indonesia, it was also demonstrated that families with low income and financial or work burdens were more vulnerable to psychosocial functioning problems, leading to child maladjustment [47]. A number of studies showed that parental financial stress due to the COVID-19 lockdown related to parental mental health, which was finally significantly associated with child and adolescent psychological well-being [48,49]. Low socioeconomic status signifies more vulnerability in several aspects, as it means less opportunity to access adaptive resources, such as safe outdoor activities and healthy food, and also less access to the healthcare system [50,51,52]. It should be noted that the disease outbreak made inequality more evident in many countries, including Thailand [53,54].

As expected, sleep problems were associated with parental stress, emotional problems, conduct problems, hyperactivity, peer problems, and all family functioning subscales. MacKenzie et al. revealed that parental stress and child sleep problems during the COVID-19 pandemic were bidirectionally correlated. Qualitative interviews showed that children had a harder time getting to sleep in less structured homes, which implied poor family functioning [55]. Wang et al. also demonstrated the association between sleep problems and harsh parenting (physical punishment and scolding) in preschool-aged children during the COVID-19 pandemic [56]. In addition, another study in China showed that children and adolescents’ sleep patterns were associated with their mental health during the pandemic [57].

Previous studies showed that the COVID-19 pandemic affects child feeding and eating behaviors [58,59,60,61]. It has been shown for a long time that eating problems in children and adolescents are closely linked to the family environment, and this was more apparent in the COVID-19 pandemic [62]. A study in the Netherlands revealed significantly higher perceived stress among parents of young children with feeding and eating problems and disorders. More difficult eating behavior by the child and more negative behavior between family members were reported [63]. In our study, we found that 50.4% of the children decreased their appetite, while 38.7% of them increased their appetite. Surprisingly, appetite change is negatively correlated to parental stress, family communication, family difficulty, peer problem change, and conduct problem change. This seemed to be contrasted with previous studies that revealed parental stress associated with child problematic eating behaviors [64]. However, it cannot be concluded that appetite change was a problem. Further studies about details in eating/feeding behaviors and children’s weight loss/gain are needed before making a conclusion.

It should be noted that Thai people had the highest scores of stress, anxiety, and depression compared to people in China, Iran, Malaysia, Pakistan, the Philippines, and Vietnam [65]. Surprisingly, family difficulty is the only domain found to be directly correlated with parental stress. However, family strength was associated with sleep problems, which finally correlated to parental stress. Family communication was associated with both sleep problems and appetite changes that were also linked to parental stress. In conclusion, this work showed the positive correlation between parental stress and family functioning in Thai families.

There are several limitations in this study. First, recall bias is difficult to avoid and there were no previous data before the COVID-19 pandemic for comparison. Second, selection bias is also possible since we asked parents who have many children to select one child to complete the questionnaire. Third, the results of this study might not represent the average Thai population since 60% of the children in this study studied in private schools, while only approximately 20% of the Thai children attended in private schools [66]. The project was announced via webpages providing childcare knowledge, so it was likely that parents who participated in this study were of high or middle socioeconomic status and well-educated.

## 5. Conclusions

This study showed increased emotional and behavioral problems in Thai children during the COVID-19 pandemic. Parental stress and family functioning were associated. Both of them were also correlated with many factors resulting from routine change. The important associated factors involved family income, screen time, physical activities, appetite change, and sleep problems. We suggest that safe outdoor activities should be easier to access in both geographical and economic aspects. It was important to note that younger children were more sensitive to the impact of the pandemic. Moreover, financial help was essential for low-income families.

## Figures and Tables

**Table 1:** Demographic data of participants (*N* = 942).

Demographic	*n* (%) or Mean [SD]
Caregiver characteristics	
Father	46 (4.9)
Mother	837 (88.9)
Others	59 (6.2)
Region (missing = 1)	
Central	687 (73.0)
North	118 (12.5)
Northeast	55 (5.8)
South	81 (8.6)
Education level	
Undergraduate	111 (11.8)
Bachelor’s degree	540 (57.3)
Higher degree	291 (30.9)
Household income (USD/month) ^a^	
<300	31 (3.3)
300–900	236 (25.1)
901–1500	233 (24.7)
1501–3000	221 (23.5)
>3000	221 (23.5)
Sex of child	
Male	491 (52.1)
Female	451 (47.9)
Type of schools	
Private	567 (60.2)
Government	263 (27.9)
Demonstrate	57 (6.1)
International	50 (5.3)
Psychiatric diagnosis of child	
Yes	151 (16.0)
No	791 (84)
Age of children (years) [Min = 5, Max = 15]	8.54 [1.87]

*n*, frequency; %, percentages; SD, standard deviation. ^a^ 1 USD is approximately 33 Thai baht.

**Table 2:** Effects of COVID-19 pandemic on children.

Effects	*n* (%) or Mean [SD]
COVID-19 infection	
Child infected	1 (0.1)
Parent infected	1 (0.1)
Child quarantined due to being in a high-risk group	13 (1.4)
Parent quarantined due to being in high-risk group	21 (2.2)
Sleep problems	
The problem has just presented or with more severity	243 (25.8)
The problem presented before and with equal severity	113 (12.0)
Problem presented before but with less severity	9 (1.0)
Appetite	
Decrease	475 (50.4)
Increase	365 (38.7)
Same	102 (10.8)
Physical activities	
<1 h/day	548 (58.2)
1–2 h/day	303 (32.2)
>2 h/day	91 (9.7)
Recreational activities	
<1 h/day	292 (31.0)
1–2 h/day	351 (37.3)
>2 h/day	299 (31.7)
Family time	
<1 h/day	340 (36.1)
1–2 h/day	337 (35.8)
>2 h/day	265 (28.1)
Screen time during COVID-19 pandemic	
Increased from before pandemic	811 (86.1)
Decreased from before pandemic	101 (10.7)
Same as before pandemic	30 (3.2)
Duration of screen time	
<3 h/day	291 (30.9)
3–5 h/day	297 (31.5)
>5 h/day	213 (22.6)
>7 h/day	141 (15.0)
Online classes	
No	149 (15.8)
1–3 h/day	480 (50.9)
3–5 h/day	238 (25.3)
5–7 h/day	75 (8)
Parental supervision during screen time	
No	48 (5.1)
Sometimes	278 (29.5)
Often	356 (37.8)
Always	260 (27.6)

*n*, frequency; %, percentages; SD, standard deviation.

**Table 3:** Strengths and Difficulties Questionnaire Scale, before and after the spread of COVID-19 (*N* = 942).

SDQ Scale (Range)	*n* (%) or Mean [SD]		*p*-Value ^a^
	Before	After	
Total difficulties (0–40)	10.98 [4.81]	12.90 [5.88]	<0.001 *
Normal (0–15)	776 (82.4%)	650 (69.0%)	<0.001 *
Borderline (16–18)	106 (11.3%)	134 (14.2%)	
Abnormal (19–40)	60 (6.4%)	158 (16.8%)	
Emotional problems (0–10)	2.04 [1.69]	2.60 [2.13]	<0.001 *
Normal (0–4)	869 (92.3%)	770 (81.7%)	<0.001 *
Borderline (5)	41 (4.4%)	70 (7.4%)	
Abnormal (6–10)	32 (3.4%)	102 (10.8%)	
Conduct problems (0–10)	2.05 [1.41]	2.53 [1.75]	<0.001 *
Normal (0–3)	809 (85.9%)	698 (74.1%)	<0.001 *
Borderline (4)	92 (9.8%)	115 (12.2%)	
Abnormal (5–10)	41 (4.4%)	129 (13.7%)	
Hyperactivity (0–10)	3.93 [2.12]	4.46 [2.42]	<0.001 *
Normal (0–5)	754 (80.0%)	653 (69.3%)	<0.001 *
Borderline (6)	81 (8.6%)	94 (10.0%)	
Abnormal (7–10)	107 (11.4%)	195 (20.7%)	
Peer problems (0–10)	2.95 [1.61]	3.30 [1.72]	<0.001 *
Normal (0–4)	787 (83.5%)	715 (75.9%)	<0.001 *
Borderline (5)	93 (9.9%)	126 (13.4%)	
Abnormal (6–10)	62 (6.6%)	101 (10.7%)	
Prosocial behavior (0–10)	6.96 [1.89]	6.82 [1.98]	<0.001 *
Normal (5–10)	873 (92.7%)	846 (89.8%)	<0.001 *
Abnormal (0–4)	69 (7.3%)	97 (10.2%)	

*n*, frequency; %, percentages; SD, standard deviation; ^a^ *p*-values derived from Fisher’s exact test for categorical variables and independent t-test for continuous variables; * *p*-value < 0.05.

**Table 4:** Results of the path analysis to examine associations between demographics, family functioning, children’s strengths and difficulties changes, and parental stress during the COVID-19 pandemic.

Variables	*β*	(95% CI)	*p*-Value
Parental stress			
Emotional problems change	0.0773	(0.0099, 0.1446)	0.025 *
Conduct problems change	0.0208	(−0.0521, 0.0936)	0.576
Hyperactivity change	0.0633	(−0.0059, 0.1326)	0.073
Peer problems change	0.0795	(0.0183, 0.1408)	0.011 *
Family strength	0.0491	(−0.0114, 0.1095)	0.111
Family difficulty	0.2597	(0.1867, 0.3327)	<0.001 *
Family communication	−0.0364	(−0.1117, 0.0389)	0.343
Child’s age	−0.0522	(−0.1108, 0.0063)	0.080
Psychiatric diagnosis	0.0489	(−0.0077, 0.1054)	0.090
Sleep problems	0.2267	(0.1692, 0.2842)	<0.001 *
Appetite changes	−0.0655	(−0.1224, −0.0086)	0.024 *
Physical activities ≥ 1 h/day	−0.0317	(−0.0894, 0.0260)	0.282
Screen time ≥ 3 h/day	0.0472	(−0.0118, 0.1061)	0.117
Emotional problems change			
Family difficulty	0.0812	(0.0064, 0.1560)	0.033 *
Family communication	0.0077	(−0.0647, 0.0801)	0.834
Psychiatric diagnosis	0.0210	(−0.0324, 0.0743)	0.441
Sleep problems	0.1969	(0.1351, 0.2587)	<0.001 *
Appetite changes	−0.0478	(−0.1102, 0.0145)	0.133
Physical activities ≥ 1 h/day	−0.0732	(−0.1355, −0.0109)	0.021*
Recreational activities ≥ 1 h/day	−0.0241	(−0.0851, 0.0370)	0.439
Screen time ≥ 3 h/day	0.0467	(−0.0146, 0.1081)	0.136
Conduct problems change			
Family strength	0.0417	(−0.0147, 0.0981)	0.147
Family difficulty	0.0413	(−0.0303, 0.1129)	0.259
Family communication	0.0070	(−0.0610, 0.0749)	0.841
Sleep problems	0.1284	(0.0659, 0.1909)	<0.001 *
Appetite changes	−0.0894	(−0.1516, −0.0272)	0.005 *
Physical activities ≥ 1 h/day	−0.0096	(−0.0735, 0.0543)	0.769
Recreational activities ≥ 1 h/day	−0.0497	(−0.1159, 0.0165)	0.141
Family time ≥ 1 h/day	−0.0812	(−0.1424, −0.0199)	0.009 *
Screen time ≥ 3 h/day	0.0927	(0.0311, 0.1543)	0.003 *
Hyperactivity change			
Family strength	0.0707	(0.0134, 0.1280)	0.016 *
Family difficulty	0.0054	(−0.0544, 0.0652)	0.860
Sleep problems	0.1515	(0.0893, 0.2138)	<0.001 *
Appetite changes	−0.0408	(−0.1033, 0.0217)	0.201
Physical activities ≥ 1 h/day	−0.0505	(−0.1144, 0.0134)	0.121
Recreational activities ≥ 1 h/day	−0.0186	(−0.0859, 0.0487)	0.587
Family time ≥ 1 h/day	−0.1037	(−0.1678, −0.0396)	0.002 *
Screen time ≥ 3 h/day	0.0821	(0.0199, 0.1443)	0.010 *
Peer problems change			
Sleep problems	0.0944	(0.0312, 0.1577)	0.003 *
Appetite changes	−0.0645	(−0.1280, −0.0011)	0.046 *
Physical activities ≥ 1 h/day	−0.1118	(−0.1743, −0.0493)	<0.001 *
Prosocial behavior change			
Income > 900 USD	0.0960	(0.0404, 0.1516)	0.001 *
Child’s age	−0.0916	(−0.1502, −0.0331)	0.002 *
Sleep problems	−0.0566	(−0.1195, 0.0062)	0.077
Appetite changes	0.0610	(−0.0018, 0.1239)	0.057
Physical activities ≥ 1 h/day	0.0918	(0.0273, 0.1563)	0.005 *
Recreational activities ≥ 1 h/day	0.0896	(0.0221, 0.1572)	0.009 *
Family time ≥ 1 h/day	0.0232	(−0.0443, 0.0906)	0.500
Screen time ≥ 3 h/day	−0.0048	(−0.0682, 0.0586)	0.882
Family strength			
Income > 900 USD	−0.1091	(−0.1710, −0.0473)	0.001 *
Sleep problems	0.0998	(0.0372, 0.1624)	0.002 *
Appetite changes	−0.0362	(−0.0995, 0.0270)	0.261
Physical activities ≥ 1 h/day	−0.0476	(−0.1087, 0.0134)	0.126
Recreational activities ≥ 1 h/day	−0.1131	(−0.1794, −0.0468)	0.001 *
Family time ≥ 1 h/day	−0.0220	(−0.0890, 0.0450)	0.521
Screen time ≥ 3 h/day	0.0234	(−0.0402, 0.0869)	0.471
Family difficulty			
Income > 900 USD	−0.1486	(−0.2092, −0.0880)	<0.001 *
Child’s age	−0.0297	(−0.0792, 0.0199)	0.241
Psychiatric diagnosis	0.0423	(−0.0061, 0.0907)	0.087
Sleep problems	0.1856	(0.1247, 0.2465)	<0.001 *
Appetite changes	−0.0742	(−0.1363, −0.0121)	0.019 *
Recreational activities ≥ 1 h/day	−0.0346	(−0.0843, 0.0151)	0.173
Family communication			
Income > 900 USD	−0.0784	(−0.1409, −0.0159)	0.014 *
Sleep problems	0.1371	(0.0745, 0.1996)	<0.001 *
Appetite changes	−0.0636	(−0.1270, −0.0003)	0.049 *
Screen time ≥ 3 h/day	0.0528	(0.0027, 0.1028)	0.039 *
RMSEA			0.017
SRMR			0.019
CFI			0.995
TLI			0.985

*β*, standardized coefficients; CI, confidence interval; RMSEA, the root mean square error index of approximation; SRMR, the standardized root mean square residual; CFI, the comparative fit index; TLI, the Tucker–Lewis index; * *p*-value < 0.05.

## Data Availability

De-identified raw data supporting the conclusions of this article will be made available by the authors on reasonable request.

## Supplementary Materials

The following supporting information can be downloaded at: https://www.mdpi.com/article/10.3390/bs14040270/s1, Table S1: The Pearson correlation coefficients of study variables.
