# Supplementary material for: Association between Family Functioning, Child Emotional and Behavioral Problems, and Parental Stress during the COVID-19 Pandemic in Thailand
Source: Behav Sci (Basel). 2024 Mar 24;14(4):270. doi: 10.3390/bs14040270 (PMC11047722; doi:10.3390/bs14040270)
Supplement: Supplementary file 1 [file behavsci-14-00270-s001.zip › behavsci-2875906-supplementary.pdf]

Table S1. The Pearson correlation coefficients of study variables.

Variables	1	2	3	4	5	6	7	8	9	10	11	12	13	14	15	16	17	18
**1. Parental stress**	1																	
**2. Emotional problems change**	0.2500*	1																
**3. Conduct problem change**	0.2126*	0.4918*	1															
**4. Hyperactivity change**	0.2141*	0.4066*	0.5587*	1														
**5. Peer problems change**	0.1819*	0.3481*	0.3261*	0.2946*	1													
**6. Prosocial behavior change**	-0.0434	-0.2625*	-0.3707*	-0.3724*	-0.304*	1												
**7. Family strength**	0.1632*	0.0595	0.0856*	0.1002*	-0.0102	0.0005	1											
**8. Family difficulty**	0.3355*	0.1446*	0.1156*	0.0810*	0.0394	-0.0574	0.2877*	1										
**9. Family communication**	0.2101*	0.0950*	0.0872*	0.0614	0.0058	-0.0326	0.3641*	0.6446*	1									
**10. Income**	-0.0549	-0.045	-0.0334	0.0177	0.0197	0.0936*	-0.1177*	-0.1558*	-0.0839*	1								
**11. Child's age**	-0.0696*	-0.0408	-0.0486	-0.029	-0.0564	-0.0829*	-0.0079	-0.0711*	-0.0606	0.0185	1							
**12. Psychiatric diagnosis**	0.1147*	0.0714*	0.0571	0.0481	0.0125	-0.023	0.0605	0.0669*	0.0206	0.0372	0.0676*	1						
**13. Sleep problems**	0.3463*	0.2335*	0.1692*	0.1818*	0.1108*	-0.0736*	0.1176*	0.2071*	0.1507*	-0.0114	-0.0553	0.1392*	1					
**14. Appetite change**	-0.1558*	-0.0965*	-0.1343*	-0.0875*	-0.0843*	0.0952*	-0.0737*	-0.1147*	-0.0913*	0.0613	-0.044	-0.0619	-0.1459*	1				
**15. Physical activities**	-0.0802*	-0.0999*	-0.0693*	-0.1115*	-0.1211*	0.1305*	-0.0688*	0.0025	0.0086	-0.0254	-0.1590*	-0.0537	-0.0622	0.0531	1			
**16. Recreational activities**	-0.0375	-0.0754*	-0.1441*	-0.1234*	-0.0488	0.1533*	-0.1566*	-0.0663*	-0.0518	0.0521	-0.2061*	-0.0387	-0.0508	0.0915*	0.1356*	1		
**17. Family time**	-0.0385	-0.0389	-0.1343*	-0.1518*	-0.0185	0.1105*	-0.1064*	-0.0439	-0.0442	0.0227	-0.2131*	0.0151	-0.0469	0.0938*	0.2698*	0.4378*	1	
**18. Screen time**	0.1006*	0.0980*	0.1551*	0.1453*	0.0619	-0.0893*	0.0846*	0.0511	0.0846*	-0.0025	0.2324*	0.0416	0.0686*	-0.0555	-0.1690*	-0.2842*	-0.2059*	1

* Significant association between variable derived from the correlation analysis.
